# Two-Axis Metabolomic Organization in the *Cistanche deserticola* Succulent Stem

**DOI:** 10.3390/plants15213465

**Published:** 2026-11-06

**Authors:** Jing Guo, Jingyi Xu, Yin Zhu, Qiang Cai

**Affiliations:** 1 Yangtze Delta Region Institute of Tsinghua University, Zhejiang, 705 Yatai Road, Jiaxing 314006, China; guojing6496@126.com; 2 School of Laboratory Medicine and Bioengineering, Hangzhou Medical College, No. 8 Yikang Street, Lin’an District, Hangzhou 311399, China

**Keywords:** *Cistanche deserticola*, spatial metabolomics, phenylethanoid glycosides, tissue distribution, medicinal plant quality, weighted coexpression network

## Abstract

*Cistanche deserticola* is a holoparasitic medicinal plant that has lost the ability to photosynthesize and grows entirely underground, so its reduced genome might suggest weak tissue-level metabolic partitioning. Using untargeted metabolomics of 132 samples from 22 plants, with each stem divided by tissue type (outer fraction vs. internal tissues) and longitudinal position (upper, middle, lower), we found a clear two-axis organization. Tissue type accounted for the smallest proportion of variance (15.2%) yet yielded the clearest discrimination: phenylethanoid glycosides (PhGs) and flavonoids were enriched in the outer fraction, whereas iridoids and primary metabolites predominated in internal tissues; along the stem, the upper part was an active metabolic zone and the lower part a sugar sink. The decline in PhG abundance with plant size occurred predominantly within tissues, with anatomical dilution contributing only 18.6%. Pathway-resolved mapping showed that the downstream steps of PhG assembly (acylation, rhamnosylation, hydroxylation, glucosylation) are progressively enriched in the outer fraction, so the discarded outer fraction is consistent with a possible site of biosynthesis, rather than merely a storage site, for acteoside and echinacoside. This stable organization likely reflects developmental canalization, indicating that gene loss does not eliminate tissue-level metabolic specialization.

## 1. Introduction

*Cistanche deserticola* Y.C.Ma (Orobanchaceae) is a holoparasitic plant cultivated for medicinal purposes in arid northwestern China. It has completely lost its photosynthetic capacity and relies on its host, *Haloxylon ammodendron*, for all nutrients [1]. The medicinal material comprises the underground succulent stem, which lacks true leaves and chlorophyll. A chromosome-level genome assembly revealed that *C. deserticola* has lost many photosynthesis-related genes but retains many transporters and carbon-metabolism genes involved in the utilization of host-derived nutrients [1]. These genomic findings have supported the interpretation that parasitic plants are characterized primarily by gene loss, and that the spatial organization they retain is a passive remnant of autotrophic ancestry [1,2].

In autotrophic plants, secondary metabolites are unevenly distributed among organs and tissues. Flavonoids accumulate in outer tissues, and alkaloids accumulate in roots or seeds [3,4]. Such spatial heterogeneity reflects functional differences among tissues [4]. If gene loss is the dominant process, a plant that has ceased photosynthesis and grows underground should exhibit little or no tissue-type metabolic partitioning. Another reason to expect weak partitioning is the subterranean environment: underground plants lack exposure to UV, transpiration stress, and above-ground herbivores. These are the principal drivers of metabolite spatial separation in autotrophs [4]. If these above-ground pressures determine tissue-level metabolic patterns, a holoparasite growing below ground should lack the usual defense–storage partition. Neither prediction has been tested using spatially resolved metabolomics.

Several studies have documented spatial heterogeneity in *Cistanche*. A ^1^H-NMR-based analysis found that primary metabolite profiles differ between the upper and lower parts of the succulent stem [5]. The total abundance of PhGs decreases sharply from the base to the apex of flowering stems [6]. The basal region is a site of active PhG glycosylation near the haustorium [7], and stems and inflorescences are enriched in distinct pathways [8]. However, no study has systematically assessed the radial and longitudinal axes within the same analytical framework, and the confounding influence of plant size on tissue composition has not been examined. The relative importance of tissue identity, longitudinal position, and morphological scaling has therefore not been resolved.

We addressed these questions in *C. deserticola*. The succulent stem has a simple anatomy that allows longitudinal division into upper, middle, and lower parts, and transverse division into the outer fraction (including the bases of the fleshy scale leaves) and internal tissues. The complete biosynthetic pathway of echinacoside has recently been reconstituted in yeast [9,10], providing a molecular basis for the interpretation of spatial patterns. We examined 22 cultivated individuals spanning a range of sizes. We divided each stem into three longitudinal parts and two transverse layers (132 samples in total) and analysed them by LC-MS/MS-based untargeted metabolomics combined with PCA, OPLS-DA, linear mixed-effects models, WGCNA, KEGG enrichment, and pathway-resolved mapping of the PhG biosynthetic route. The analysis focused on three questions: whether metabolites vary systematically among tissue types and longitudinal positions; whether the size-related decline in outer-fraction ratio represents an independent source of variation or is secondary to tissue type; and whether the spatial patterns align with known biosynthetic functions, particularly the location of the terminal steps of PhG assembly.

## 2. Results

### 2.1. Metabolomics Data Overview and Quality Control

LC-MS/MS analysis of 132 samples from 22 *C. deserticola* plants, covering three longitudinal regions and two tissue layers, yielded 17,201 features. After excluding features with a QC coefficient of variation > 30% and features containing rare elements (F, Si, Cl), 8619 features remained. The tight clustering of QC samples confirmed analytical reproducibility. All subsequent quantitative analyses report log2-transformed relative abundance (semi-quantitative, PQN-normalized); these values do not indicate absolute concentrations (mg/g DW). 

### 2.2. Size Effects on PhG Abundance Operate Mainly Within Tissues, with Anatomical Dilution as a Minor Component

**Figure 1:**
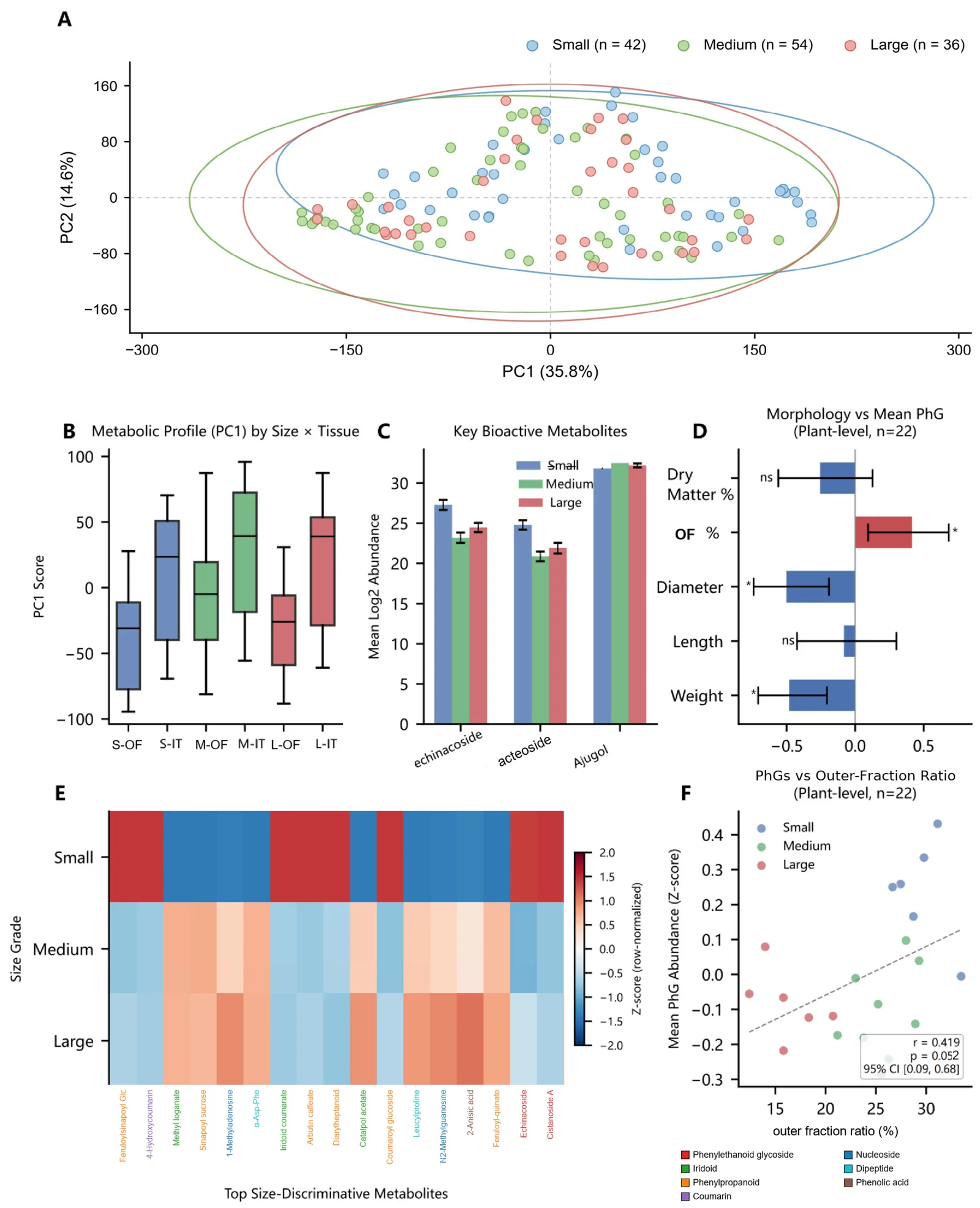
Association between *C. deserticola* size grade and relative feature abundance. (**A**), PCA score plot of PC1 versus PC2 (PC1 35.8%, PC2 14.6% of total variance); each of the three size grades is enclosed by a 95% confidence ellipse. (**B**), Boxplots of PC1 scores in the outer fraction and internal tissues of the three size grades. Boxes indicate the interquartile range (IQR), the central line indicates the median, and whiskers show the range (1.5 × IQR); one-way ANOVA, where *n* = 7, 9, and 6 plants in the small, middle, and large grades, respectively. (**C**), Relative abundances of representative PhGs across the three size grades. Bars indicate mean abundance following probabilistic quotient normalization and log_2_ transformation (mean log_2_ abundance); error bars represent mean ± SD; *n* = 7, 9, and 6 biological replicates (plant means) in the small, middle, and large grades, respectively. Small, blue; middle, green; and large, red. (**D**), Strength of correlations between morphological indicators of *C. deserticola* and individual mean PhG abundance (Pearson r with bootstrap 95% CI; plant-level, *n* = 22). * *p* < 0.05; ns, not significant (*p* ≥ 0.05). Mean PC1 correlates with PhG relative abundance (mean PC1 vs. mean PhG: r = −0.686, *p* < 0.001), but this association reflects the shared longitudinal-position signal rather than PhG-specific variation; PC1 is therefore not a specific proxy for PhG abundance. (**E**), Heatmap of the 15 most size-discriminative confidently identified metabolites (Level 2; stratified ANOVA within tissue–position subgroups) plus the signature phenylethanoid glycosides echinacoside and cistanoside A (Level 1). Columns are labelled with metabolite names and coloured by compound class (see legend). (**F**), Positive correlation between mean PhG abundance and the outer fraction ratio calculated from the plant-level aggregated data (Pearson r = 0.419, 95% bootstrap CI [0.09, 0.68], *p* = 0.052), with each point representing an individual plant mean (*n* = 22), coloured by size grade (small, blue; middle, green; large, red); the dashed line represents the least-squares regression line.

**Figure 2:**
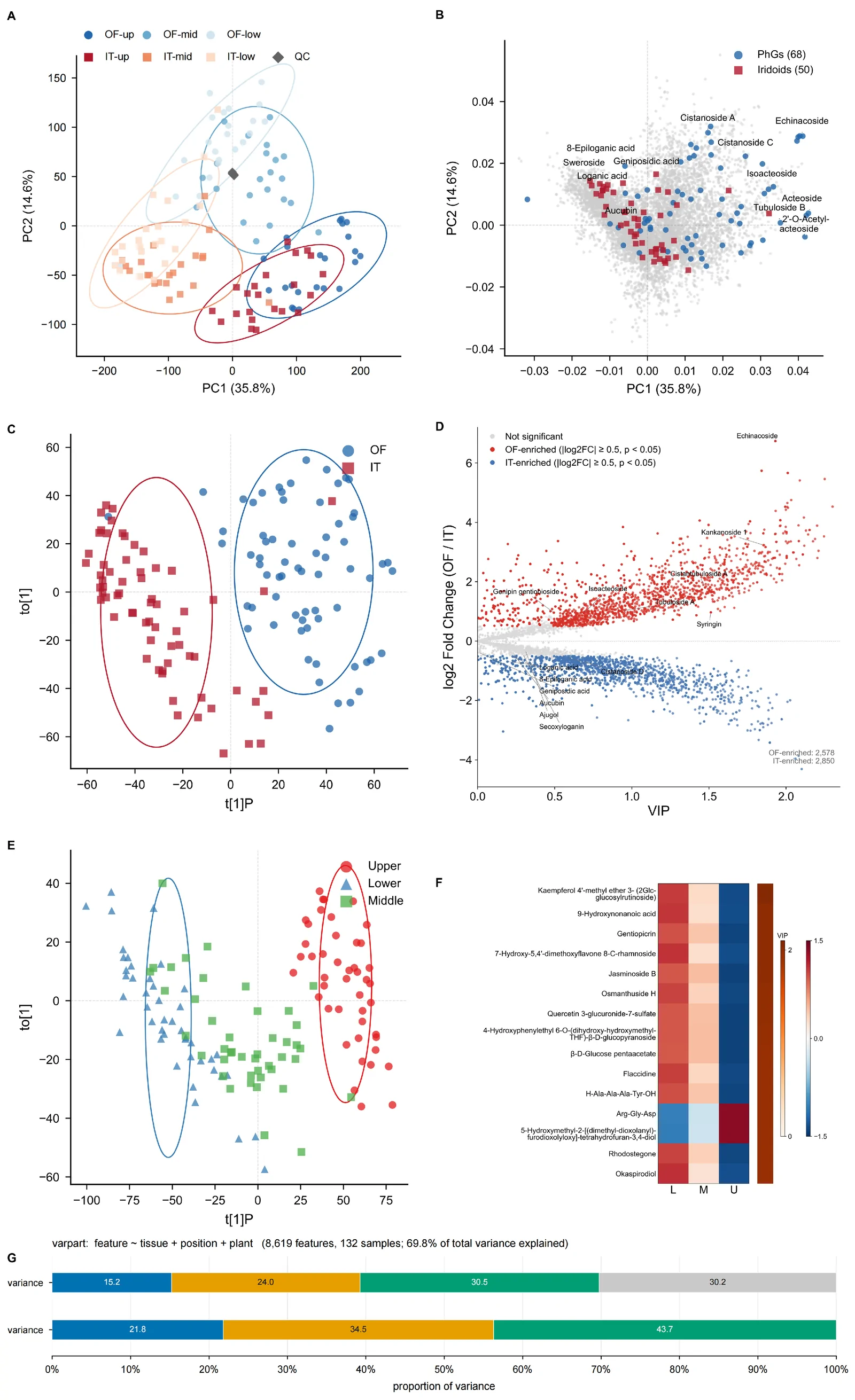
Multidimensional metabolomic profiling across *C. deserticola* stem tissues and positions. (**A**), PCA score plot of PC1 versus PC2. PC1 (35.8%) arranges samples according to longitudinal position; PC2 (14.6%) separates the outer fraction from internal tissues. In each ellipse encloses the 95% confidence region for the corresponding group. (**B**), PCA loading plot for PC1 and PC2; phenylethanoid glycosides (echinacoside, acteoside, isoacteoside) have the largest positive loadings, whereas iridoid glycosides (loganic acid, aucubin) lie near zero or on the negative side. (**C**), OPLS-DA score plot comparing the outer fraction with internal tissues (R^2^X = 0.346, R^2^Y = 0.750; GroupKFold Q^2^ = 0.587; 200 permutations, *p* < 0.001). (**D**), Volcano plot comparing the outer fraction with internal tissues (VIP vs. log2 fold change, OF/IT; |log2FC| ≥ 0.5, *p* < 0.05; 2578 features enriched in the outer fraction, 2850 in internal tissues). (**E**), OPLS-DA score plot comparing samples from upper and lower positions (R^2^X = 0.198, R^2^Y = 0.960; GroupKFold Q^2^ = 0.491; 200 permutations, *p* < 0.001). (**F**), Heatmap of 15 Cistanche-associated differential metabolites (VIP > 1.9, nominal *p* < 1 × 10^−20^) across lower, middle, and upper groups; colors represent Z-score-normalized mean log2 abundance, and the bar on the right indicates VIP. (**G**), Variance partitioning of the feature matrix (feature ~ tissue + position + plant). The two bars give the variance fractions as a percentage of the total variance and as a percentage of the explained variance; the three factors together explain 69.8% of the total variance.

The 22 plants were assigned to small, middle, and large size grades (Table A1). PCA using all features showed no separation among size-grade groups (Figure 1A,B). Mean PC1 was significantly correlated with mean PhG abundance (r = −0.686, *p* < 0.001; Figure 1D); however, because PC1 is dominated by longitudinal position rather than tissue identity (Figure 2A), this indirect correlation does not establish PC1 as a specific indicator of PhG abundance. Across grades from small to large, plant weight increased approximately 4.3-fold, while the outer fraction ratio fell from 29.2% to 16.2%; the dry matter ratio was unchanged (Table 1). Thus, growth largely reflects expansion of internal tissues, which reduces the proportional contribution of the outer fraction.

Size was related to PhG abundance through both tissue composition and within-tissue abundance, which were quantified separately below. Echinacoside, acteoside, and ajugol tended to be more abundant in the small size grade (Figure 1C,E). Mean PhG abundance was positively associated with the outer fraction ratio (r = 0.419; bootstrap 95% CI [0.09, 0.68], which excludes zero; parametric Pearson *p* = 0.052; Figure 1F) and negatively associated with weight (r = −0.49, *p* = 0.026) and diameter (r = −0.51, *p* = 0.016) at the individual plant level (*n* = 22).

To quantify the two contributions, whole-stem PhG relative abundance was reconstructed in linear space as dry-mass-weighted averages using the tissue dry masses listed in Table A1, and the size effect was decomposed counterfactually (Section 4.4). Whole-stem PhG relative abundance decreased by ~80% from small to large plants (geometric mean ratio 0.202). The composition term (the reduction in the outer-fraction dry-mass share from 29.2% to 16.2%) accounted for 18.6% (95% CI 12.8–27.2%) of the log-scale decline, and the remaining 81.4% was accounted for by lower relative abundance within tissues, consistent with the within-tissue correlations (internal tissues: r = −0.55, *p* = 0.008; outer fraction: r = −0.41, *p* = 0.06). Per-plant decomposition confirmed this ordering: the composition channel was small and stable (log-scale SD 0.238), whereas the intra-tissue channel showed 5.9-fold greater variability (SD 1.416), and whole-stem abundance tracked within-tissue outer-fraction abundance (R^2^ = 0.964) much more closely than the outer-fraction share itself (R^2^ = 0.252). Thus, the lower PhG relative abundance in large plants primarily reflects a genuine within-tissue decrease, with a modest secondary contribution from anatomical dilution. This finding challenges the common preference for large, fleshy stems: appearance-based grading by size trades off against PhG-based quality, which was highest in small-grade material.

### 2.3. Tissue Type and Longitudinal Position Define Additive Axes of Metabolic Variation

The sampling design comprising 22 plants, three positions and two tissues is fully balanced. The three factors are therefore mutually orthogonal, and all fractions of shared variance are zero by construction. Additive variance partitioning attributed 15.2% of total variance to tissue type, 24.0% to longitudinal position and 30.5% to plant identity (varpart, 999 permutations; Figure 2G). An REML random-intercept mixed model fitted to each feature and Euclidean PERMANOVA (9999 permutations) yielded the same ordering and resolved a tissue × position interaction of 2.8% of total variance (within-plant permutation, strata = plant: *p* = 0.0001). Plant identity was therefore the largest individual source of variation in metabolites. Variance shares describe the distribution of observed variation among factors and are not causal effects.

Ordination separated the two spatial factors along different components (Figure 2A). PC1 (35.8% of variance) was dominated by the longitudinal contrast (envfit R^2^ = 0.486, *p* ≤ 1 × 10^−4^), with the upper, middle and lower samples forming an ordered sequence; tissue fraction dominated PC2 (14.6%; envfit R^2^ = 0.310, *p* ≤ 1 × 10^−4^), separating the outer fraction from the internal tissues, with samples from the middle lying between the upper and lower groups. The loading plots (Figure 2B) indicated that PhGs (echinacoside, acteoside, 2′-O-acetylacteoside, isoacteoside) had the highest positive loadings on PC2 (the chemical markers of the outer fraction), whereas iridoid glycosides (loganic acid, 8-epiloganic acid, sweroside, geniposidic acid, aucubin) had loadings near zero or on the negative side, consistent with their enrichment in internal tissues.

OPLS-DA distinguished the outer fraction (*n* = 66) from internal tissues (*n* = 66) and the upper from the lower part (Figure 2C,E), with 2578 features enriched in the outer fraction and 2850 in internal tissues (Figure 2D). Because the samples are nested within plants, both comparisons were re-tested at the level of individual plants: paired within-plant comparisons confirmed the outer fraction–internal tissue separation (echinacoside paired t = 11.9, *p* = 8.9 × 10^−11^), and GroupKFold cross-validation, with all samples from each plant kept within a single fold, yielded Q^2^ = 0.587 for the tissue contrast and Q^2^ = 0.491 for the position contrast. Negative intercepts from permutation tests and significant CV-ANOVA (*p* < 10^−10^ in both cases) confirmed that the discriminant structure does not result from within-plant information leakage. Volcano plot analysis identified 3019 features with higher abundance in the upper part and 2260 in the lower part (|log2FC| > 1, *p* < 0.05), and a heatmap of 15 differential metabolites showed distinct patterns along the longitudinal axis across lower, middle and upper segments, spanning phenylethanoid glycosides, flavonoids, oligopeptides, saccharide derivatives, iridoid glycosides, hydroxy fatty acids, diterpenoid alkaloids, terpenoids and spirodiols (Figure 2F).

Considered alongside the small tissue × position interaction (2.8%), these results establish tissue type and longitudinal position as largely additive axes of metabolic organization: the outer fraction and the upper part each contain a distinct metabolite profile, while the middle position, the only one with no associated module and no extreme scores along either axis, forms a transition zone between the upper and lower metabolic programmes.

### 2.4. Key Metabolites Define the Two Axes at the Compound Level

**Figure 3:**
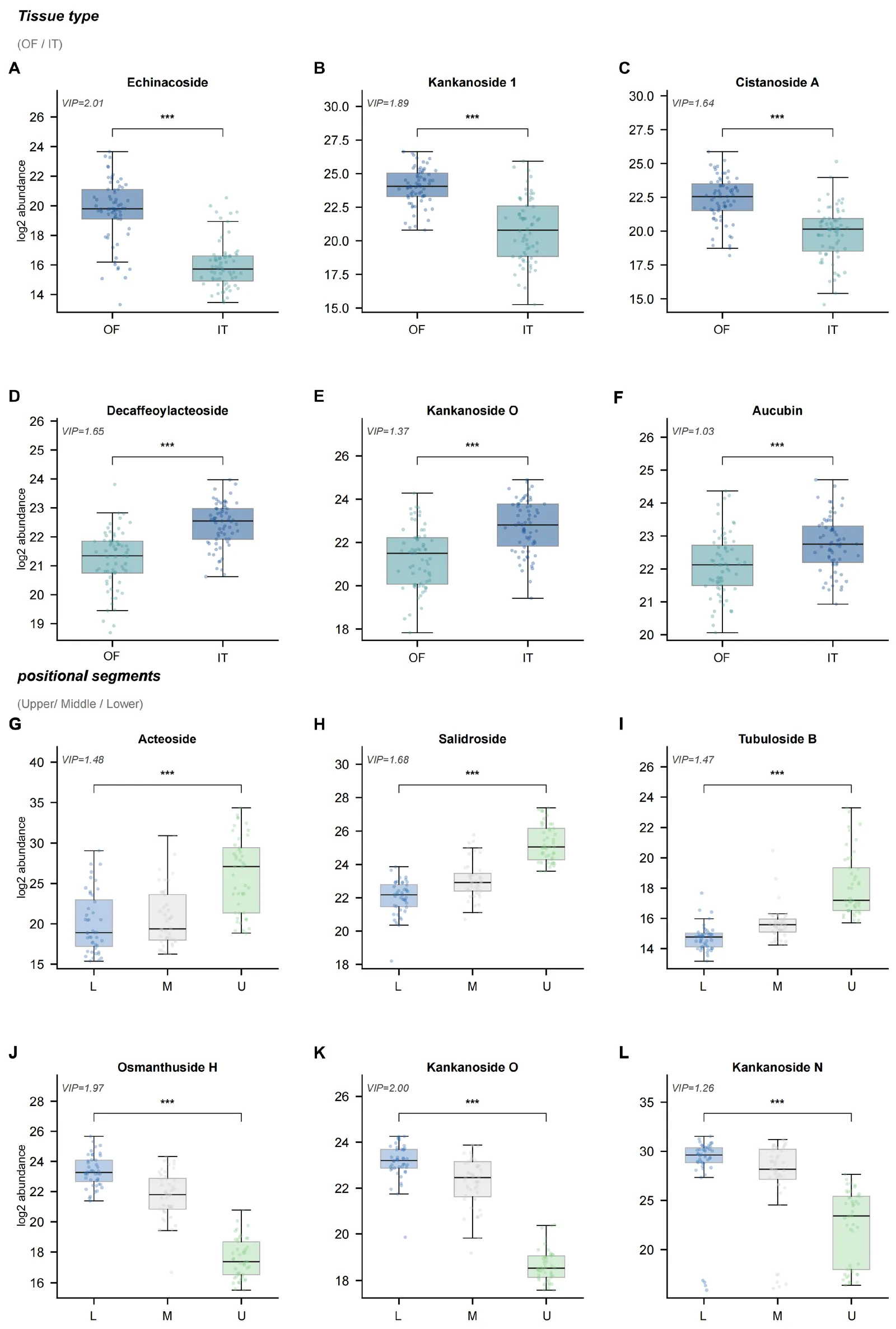
Comparison of relative abundances of representative differential features among tissue types and longitudinal positions. (**A**–**F**), Log_2_ abundance comparisons across tissue types (OF, outer fraction; IT, internal tissues); (**G**–**L**) comparisons of log_2_ abundances across longitudinal positions (L, lower; M, middle; U, upper). Boxplots show the distribution (median and quartiles) and each point represents the value for an individual biological replicate. VIP values (OPLS-DA variable importance in projection) are shown in the upper-left corner. Asterisks denote statistically significant differences between groups (*** *p* < 0.001).

After establishing the two axes statistically, we identified the metabolites associated with them (Figure 3). The longitudinal gradient was characterized by lower-enriched PhGs (e.g., osmanthuside H and kankanoside O), upper-enriched RGD-containing oligopeptides and β-D-glucose pentaacetate, and divergent flavonoid patterns. Acteoside, kankanoside 1, and cistanoside A were significantly more abundant in the outer fraction than in internal tissues, whereas decaffeoylacteoside, kankanoside O, and aucubin preferentially accumulated in internal tissues (*p* < 0.01; Figure 3A–F). Longitudinally, acteoside, salidroside, and tubuloside B had higher abundance in the upper part and lower abundance in the lower part, whereas osmanthuside H, kankanoside O, and kankanoside N were enriched in the lower part (Figure 3G–L). The lower-part enrichment of osmanthuside H, a PhG, differs from the overall upper-enriched pattern of PhGs and suggests a possible role near the parasitic interface.

### 2.5. Co-Abundance Networks Resolve the Two Axes into Metabolic Modules

**Figure 4:**
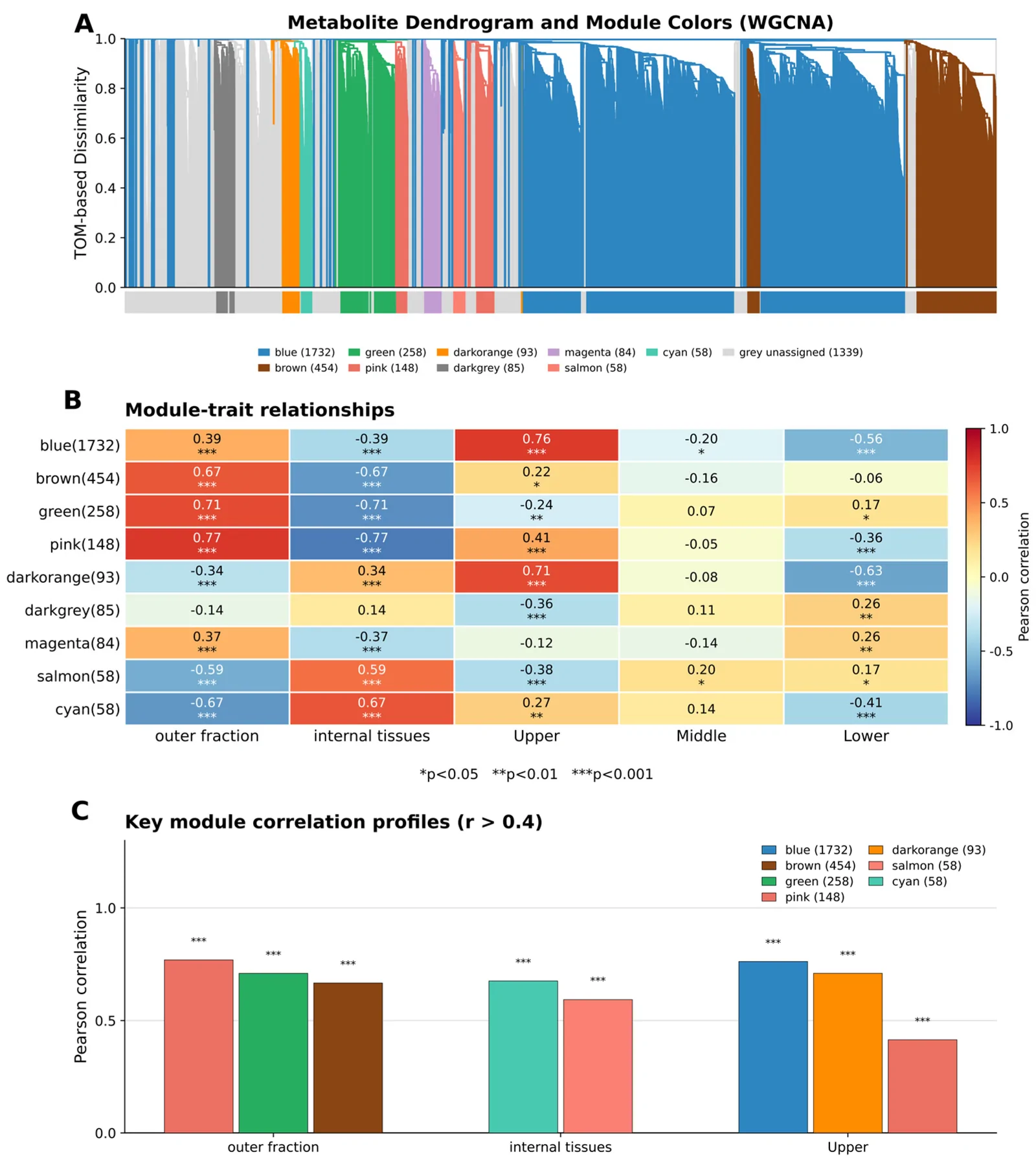
WGCNA identifies metabolite modules correlated with stem regions of *C. deserticola*. (**A**), Hierarchical clustering dendrogram of the 4309 variance-filtered metabolites based on TOM dissimilarity (average linkage), with module assignments identified by the colour bar underneath; dynamic tree cut (cut height 0.98), minimum module size 50, and eigengene merging (threshold 0.75) yielded ten modules (nine non-grey plus grey). (**B**), Module–trait relationship heatmap. Each row is a WGCNA module (with the number of metabolites in parentheses); each column is a trait (outer fraction, internal tissues, Upper, Middle, Lower). Cells show the Pearson r and significance stars; red denotes positive and blue negative correlation. Sixteen module–trait pairs reach |r| ≥ 0.4. (**C**), Correlation profiles of the key modules (|r| > 0.4) for the traits with which they are primarily associated; bar colours correspond to module names (*** *p* < 0.001).

**Figure A1:**
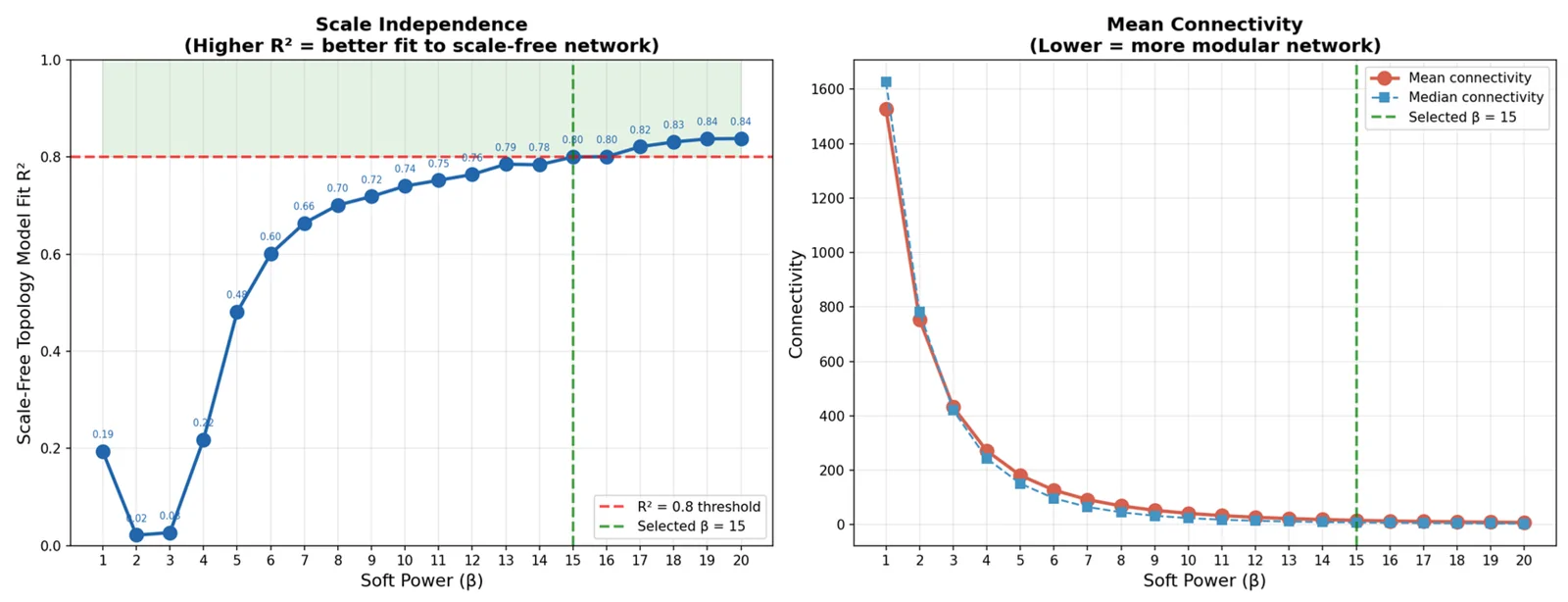
Selection of the soft-thresholding power (β) for WGCNA. (**Left**): Scale-free topology model fit (R^2^) as a function of β, with the red dashed line marking the R^2^ = 0.80 threshold; (**right**): mean connectivity as a function of β. To obtain R^2^ ≥ 0.80 while maintaining sufficiently low mean connectivity, β = 15 was selected (scale-free R^2^ = 0.8), meeting the scale-free network topology requirement.

Hierarchical clustering separated samples first by tissue type and subsequently by longitudinal position (Figure 4A). WGCNA was performed on the 4309 metabolites remaining after variance filtering (top 50%, threshold 2.5443). The selected soft-thresholding power was β = 15, the lowest power that attained a scale-free topology fit of R^2^ ≥ 0.8 (R^2^ = 0.8004; Figure A1). The network resolved ten modules, nine non-grey plus grey, covering 2970 assigned metabolites (68.9% of the filtered set). The blue module was substantially larger than the others (1732 metabolites, 40.2%), followed by brown (454), green (258) and pink (148); the smallest non-grey modules, cyan and salmon, each contained 58 metabolites; and the grey module (1339, 31.1%) comprised unassigned features and was excluded from subsequent enrichment analyses.

Module–trait correlations (Figure 4B,C) identified 16 significant pairs (|r| ≥ 0.4). Three modules showed an outer-fraction-dominant pattern, namely pink (r = +0.769, *p* = 5.5 × 10^−27^), green (+0.709) and brown (+0.666), whereas two modules showed the inverse pattern (internal tissues), cyan (+0.675) and salmon (+0.593). Together these modules span the radial axis. Across positions along the longitudinal axis, the blue module was strongly positively correlated with the Upper position (r = +0.761, *p* = 3.1 × 10^−26^) and negatively with Lower (r = −0.560, *p* = 2.9 × 10^−12^), and darkorange showed the same direction of association (Upper +0.709, Lower −0.631). The pink module also had a dual assignment (outer fraction + Upper, r = +0.415), paralleling the interface programme identified in the ordination (Section 2.3). None of the modules reached |r| ≥ 0.4 for the Middle position.

Because the 132 samples are nested within 22 plants, the module–trait structure was validated for individual metabolites: a linear mixed-effects model (metabolite ~ Tissue × Position + (1|Plant)) was fitted to each of 101 hub metabolites. All 101 models converged: 24 with the default L-BFGS optimizer and 77 after switching to BFGS, where L-BFGS had failed to converge. The initial failures were therefore optimizer-specific and not attributable to near-zero variance; only two models showed a plant-variance component at the boundary (<1 × 10^−6^), and no model had near-zero residual variance. The models yielded 606 fixed-effect coefficients, of which 466 reached significance after Benjamini–Hochberg correction (FDR < 0.05; Supplementary Table S2). Tissue main effects were significant in 92/101 models, Lower in 96 and Middle in 91, whereas Tissue × Position interactions reached significance in only 39 (Lower) and 47 (Middle) models, again indicating largely additive axes. The top hub metabolites included Scleritoitode H/I/J/K and Penicillithiophenol A (pink) and Rhamnazin-3-rhamnoside (brown), reflecting the consistent grouping of phenylethanoid- and flavonoid-class metabolites.

### 2.6. Pathway-Level Organization: KEGG Enrichment and Pathway-Resolved Mapping

**Figure 5:**
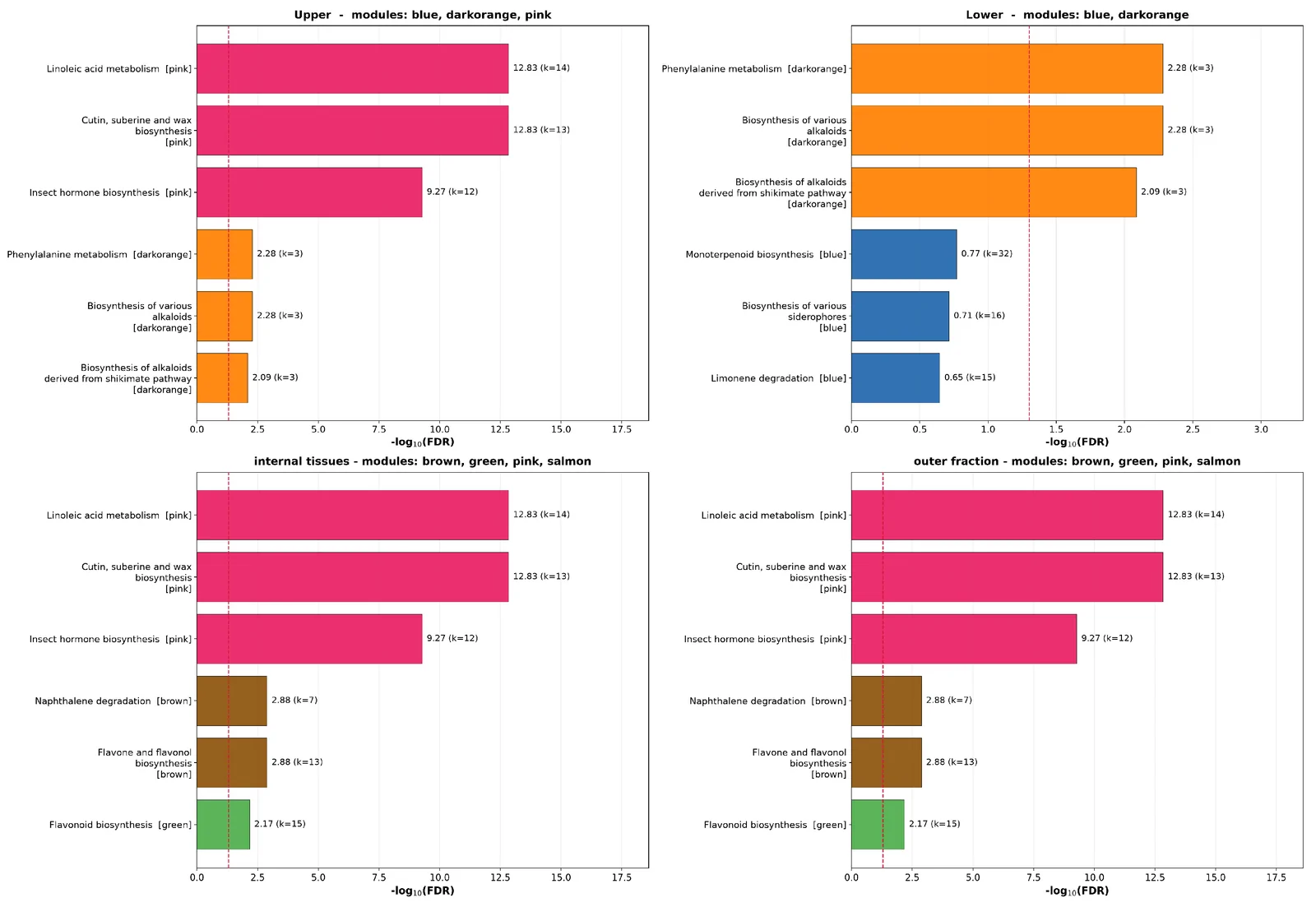
KEGG pathway enrichment of spatially partitioned metabolites in the *C*. *deserticola* succulent stem. KEGG pathway enrichment for the outer fraction (OF), internal tissues (IT), and upper and lower groups, respectively. Bars represent significantly enriched pathways ordered by −log_10_(FDR). Values beside the bars indicate the fold enrichment (FE), and the bracketed label indicates the WGCNA module to which the metabolites were assigned. Enrichment was assessed by one-sided hypergeometric tests using the universe of 331 annotated metabolites, with Benjamini–Hochberg correction across all tested pathways. Solid bars indicate FDR < 0.05, and hatched bars indicate 0.05 ≤ FDR < 0.10 (exploratory).

The metabolic patterns of the modules were then related to their biosynthetic routes. KEGG enrichment of the key modules used one-sided hypergeometric tests with Benjamini–Hochberg correction against a background of 331 pathway-annotated metabolites (692 metabolites in the key modules were mapped to KEGG compounds by molecular formula or compound name). The analysis yielded 24 significantly enriched pathway–module pairs (FDR < 0.05; Figure 5). Several pathways are mechanistically relevant. Monoterpenoid biosynthesis, the pathway leading to the iridoid glycosides characteristic of *C. deserticola*, showed nominal enrichment in the blue module (*p* = 0.0017) that did not survive correction (FDR = 0.17) and was not enriched in the green module (*p* = 0.37); linoleic acid metabolism, the source of jasmonic acid precursors, was strongly enriched (pink module, FDR = 1.5 × 10^−13^); site-resolved enrichment localized the cutin, suberine and wax biosynthesis pathway, together with linoleic and α-linolenic acid metabolism, to the outer-fraction-associated pink module (FDR = 1.5 × 10^−13^); and phenylalanine metabolism was assigned to the Upper-associated darkorange module (FE = 20.3). The convergence of monoterpenoid, linoleic acid and cuticular lipid signals with the known chemical profile of *C. deserticola* (iridoids, phenylethanoid glycosides, flavonoids) provides pathway-level validation that the co-abundance modules represent biosynthetic pathways of medicinal relevance.

**Figure 6:**
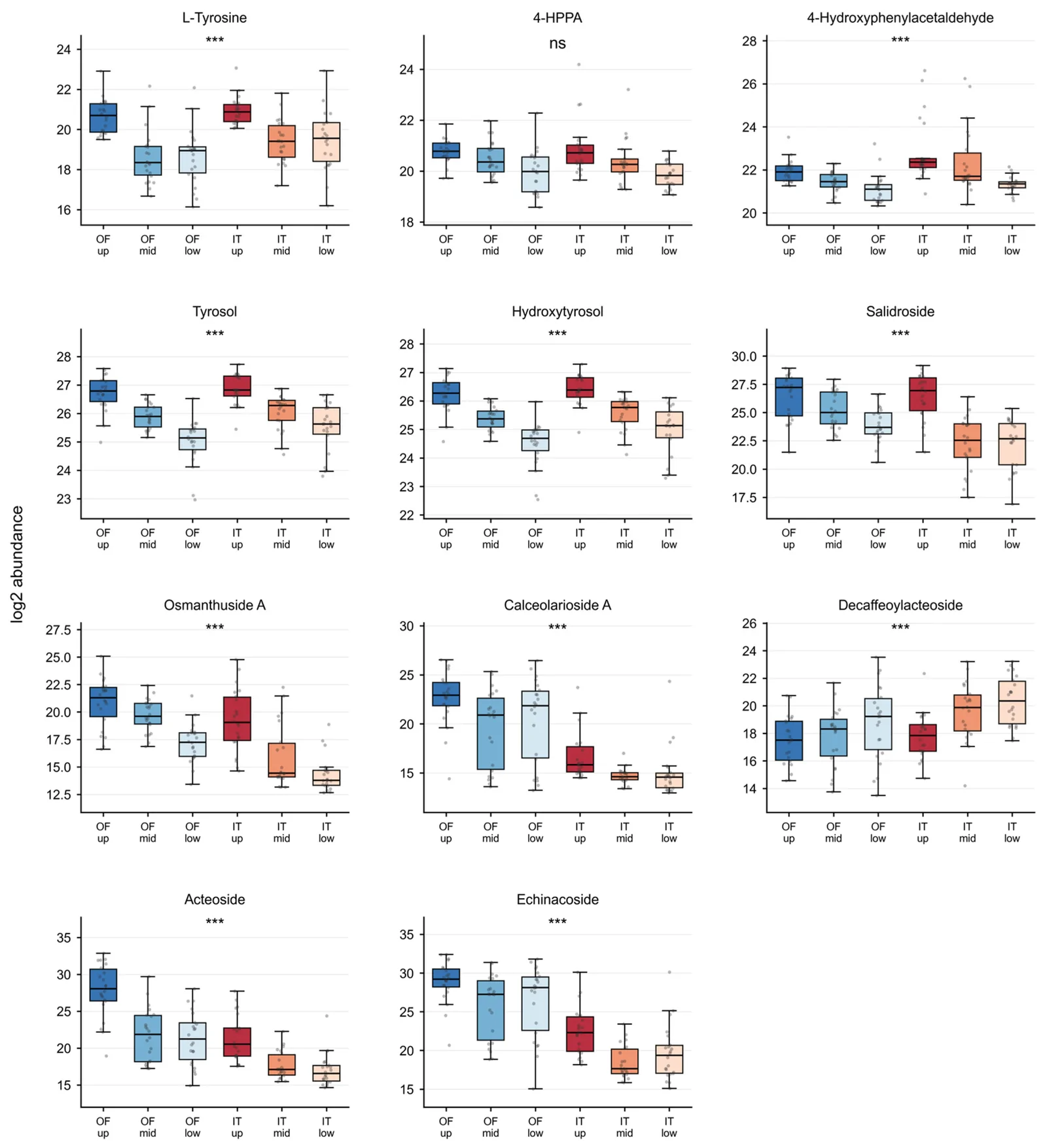
Spatial distribution of PhG pathway metabolites across the six tissue–position groups. Boxplots show the log_2_ relative abundances of eleven biosynthetic intermediates in the outer fraction (OF-upper/middle/lower) and internal tissues (IT-upper/middle/lower), arranged from upstream to downstream along the reconstructed pathway [9,10]: L-tyrosine, 4-HPPA, 4-hydroxyphenylacetaldehyde, tyrosol, hydroxytyrosol, salidroside, osmanthuside A, calceolarioside A, decaffeoylacteoside, acteoside, and echinacoside. The ordering reflects the reported pathway rather than the observed abundances. The upstream intermediates show only small differences between tissues (|log2FC| ≤ 0.7), whereas the downstream intermediates (salidroside onward) are progressively enriched in the outer fraction (log2FC 1.6–6.8); decaffeoylacteoside, by contrast, is enriched in internal tissues. Asterisks indicate significant outer-fraction vs. internal-tissue differences (*** *p* < 0.001 ns, not significant; Wilcoxon signed-rank test on 66 within-plant pairs, 22 plants × 3 positions pooled, with Benjamini–Hochberg correction). Pooling positions is conservative: a sensitivity mixed model (metabolite ~ tissue + position + (1|plant)) and position-stratified paired tests gave the same ten significant intermediates and a non-significant 4-HPPA.

To examine these patterns in individual metabolites, the relative abundances of eleven intermediates in the phenylethanoid glycoside pathway were compared across the six tissue–position groups (Figure 6). The upstream portion of the pathway was essentially uniform: L-tyrosine, 4-HPPA, 4-hydroxyphenylacetaldehyde, tyrosol and hydroxytyrosol showed comparable abundances in the outer fraction and internal tissues at all three positions, indicating that early tyrosine catabolism is not spatially restricted. Downstream, the pathway showed progressively greater polarization towards the outer fraction: the outer-fraction-to-internal-tissue abundance ratio increased from ~3 for salidroside and ~7 for osmanthuside A to ~30–40 for acteoside and calceolarioside A and ~110 for echinacoside. Longitudinally, salidroside, osmanthuside A and acteoside were most abundant in the upper part. By comparison, decaffeoylacteoside, the product of an alternative branch in which rhamnosylation preceded caffeoylation, behaved differently (Figure 3), accumulating in internal tissues including the lower core. Taken together, these patterns define a spatially graded assembly route. The early steps operate throughout the stem, whereas the downstream steps (acylation, rhamnosylation, hydroxylation and final glucosylation) are progressively concentrated in the outer fraction, consistent with the pathway order characterized in *Cistanche tubulosa* [10]. This pathway-level gradient corresponds to the network assignment of the co-abundance modules. The outer-fraction-restricted intermediates fall within the outer-fraction-associated pink and green modules, whereas the internal-tissue-accumulating decaffeoylacteoside is assigned to the brown-module side of the radial partition. Two independent levels of evidence therefore converge on the same radial pattern.

## 3. Discussion

Our spatially resolved metabolomic analysis shows that *C. deserticola* retains a clear two-axis metabolic architecture despite gene loss and an underground lifestyle. Before interpreting this architecture, one statistical distinction requires consideration: tissue type accounted for the smallest variance share (15.2%) yet produced the strongest supervised discrimination (plant-independent Q^2^ = 0.587). Supervised discrimination strength and variance share address different questions. The former assesses whether a factor produces a directionally coherent signal, and the latter quantifies its share of total variation. The contrast arises because nearly all PhGs shifted coherently towards the outer fraction, whereas the larger plant and position variances are directionally heterogeneous and act as background rather than as a separable signal. Both axes showed significant variation within plants (PERMANOVA strata = plant), and the metabolite-level mixed-effects models (median plant ICC = 0.27, range 0.00–0.64; Supplementary Table S2) confirm that the pattern remains robust after accounting for between-plant variation.

Why does this organization persist without the selective pressures that shaped it in autotrophs? Developmental canalization offers the most parsimonious explanation. The *Cistanche* body plan is inherited from autotrophic ancestors and develops along radial and longitudinal axes; because epidermal and internal tissues arise from distinct developmental lineages (protoderm versus ground meristem), their metabolic divergence may result from the formation of the body plan rather than from current environmental cues. This interpretation is consistent with the four developmental domains identified here. The absence of a middle-position module marks the natural transition between them. The similarity to autotrophic relatives, despite the absence of UV, herbivory or transpiration, suggests a developmental default rather than ongoing selection; comparison with *Cistanche armena* [11] indicates the pattern may extend across the genus.

The pathway-level results show where this architecture is expressed. KEGG enrichment linked the radial programme to cuticular lipid deposition and flavonoid terminal biosynthesis, with only nominal enrichment of the iridoid precursor pathway, and linked the longitudinal programme to phenylpropanoid precursor supply; linoleic acid metabolism (the lowest-FDR pathway) implicates jasmonate signalling, a central regulator of phenylpropanoid accumulation [12]. The pathway-resolved mapping (Figure 3 and Figure 6) traced one such route through its individual metabolites, and its clearest feature is a substrate–product split. The downstream intermediates and their products (acteoside, echinacoside) were spatially enriched in the outer fraction, most strongly at the apex, whereas decaffeoylacteoside, the product of the alternative branch in which rhamnosylation preceded caffeoylation, accumulated in internal tissues. This is consistent with a model in which the outer fraction may execute the downstream acylation, rhamnosylation, hydroxylation and glucosylation steps that convert salidroside into acteoside and echinacoside, while the internal tissues retain the alternative-branch intermediate decaffeoylacteoside. This split may reflect two non-exclusive mechanisms: higher expression or activity of the corresponding glycosyltransferases [13] and BAHD acyltransferases [14,15,16] in the outer fraction, or loss of the caffeoylated products in internal tissues through esterases or absent transporters. Identifying the *Cistanche* acyltransferase, rhamnosyltransferase, hydroxylase and glucosyltransferase genes and comparing their expression in the outer fraction and internal tissues by spatial transcriptomics would directly validate this model.

Along the longitudinal axis, the gradients point to developmental processes and the parasitic interface. The upper part (apical meristem) combined secondary and primary metabolism, and the enrichment of acteoside in the upper outer fraction (Figure 6) is consistent with local phenylpropanoid biosynthesis at the apex. The lower part has a signature characteristic of a sugar sink, and its enriched PhGs (osmanthuside H, kankanoside O) may participate in haustorial signalling: parasitism alters host phenylpropanoid pathways [7] and phenolic acids induce haustorium formation [17]. The upper-enriched acteoside pattern contrasts with basal enrichment in flowering stems [6,7], reflecting differences in developmental stage; the longitudinal distribution of PhGs is thus compound- and stage-specific [2], whereas the vegetative stems examined here bore no inflorescences. Dual module assignment (pink: outer fraction + upper) is consistent with an interaction between the two axial programmes. Spatial transcriptomics at the parasitic interface could be used to test the haustorial-signalling hypothesis [17].

The discarded outer fraction is not only a major reservoir of PhGs [5,6], the bioactive constituents whose medicinal activities are well documented [18,19], but also a tissue fraction in which downstream acylation, rhamnosylation, hydroxylation and final glucosylation steps of PhG assembly are enriched (Figure 6); retaining this fraction during processing therefore preserves both the accumulated product and the tissue in which these steps are enriched. Moreover, the PhG decline in large plants was attributable only in part to anatomical dilution (18.6%, 95% CI 12.8–27.2%), arising predominantly from a genuine within-tissue decline (Section 2.2), a finding that challenges the “larger is better” assumption and helps explain the inconsistency of results of whole-stem homogenate analyses [20,21,22]: size-based grading trades off against PhG-based quality.

Several limitations qualify these conclusions. First, the sample size is modest (*n* = 22) and all abundance measures are PQN-normalized relative abundances, not concentrations; targeted quantification of the main PhGs with calibration curves is necessary before quality statements can be expressed in units of mass. Second, module boundaries were derived from co-abundance across 132 samples without validation using an independent dataset, and because modules are defined by co-abundance, module-level directional claims were not equated with abundance changes in individual metabolites; metabolite-level conclusions rest on the mixed-effects estimates. Third, because the succulent stem has no clear anatomical peel boundary, some internal tissue may have been included in the outer fraction; this admixture would reduce the radial contrasts, so the reported tissue differences are conservative. Fourth, monoterpenoid biosynthesis reached nominal significance with very few matched metabolites, and annotation coverage of the smaller modules (e.g., cyan) is low; targeted assays and improved annotation are required before these pathways can be assigned definitively. Future studies combining spatial transcriptomics with mass-spectrometry imaging at cellular resolution, and testing whether patterns differ with host identity [23], would help establish whether the developmental default remains fixed or exhibits plasticity.

## 4. Materials and Methods

### 4.1. Plant Material and Tissue Dissection

Twenty-two plants of *C. deserticola* were obtained from the Cistanche cultivation base in Gaizikumu Township, Shaya County, Aksu Prefecture, Xinjiang, China; identified by Prof. Haiyan Xu (Xinjiang Medical University); and transported immediately to the laboratory. All plants parasitized *H. ammodendron*. For each plant, we measured stem length; diameters of the upper, middle, and lower parts; and fresh weight. All stems sampled were vegetative and entirely subterranean.

**Figure 7:**
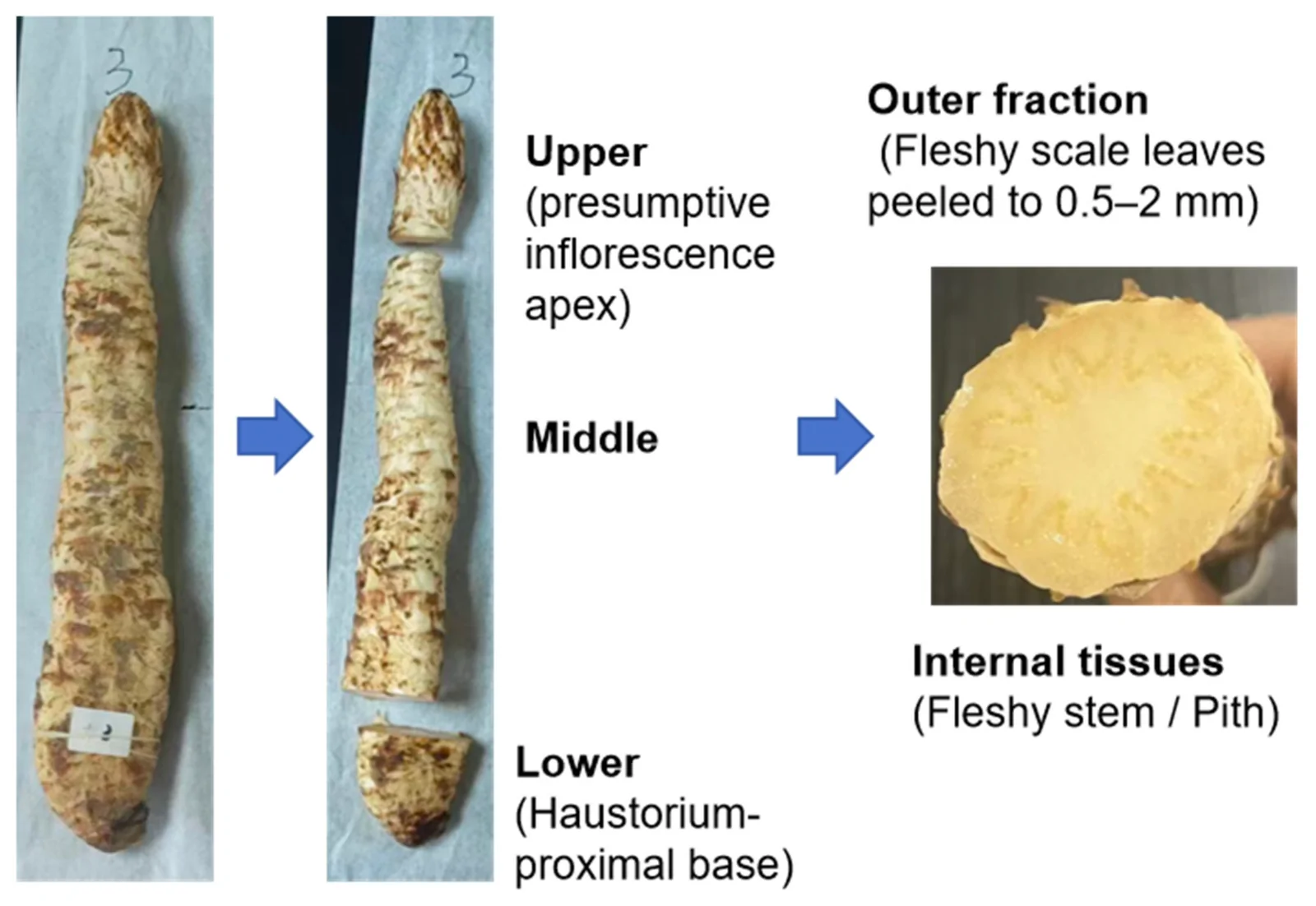
Tissue dissection scheme for *C. deserticola*. Schematic showing longitudinal (upper, middle, lower) and transverse (outer fraction and internal tissues) sampling of the succulent stem of *C. deserticola*, yielding six tissue samples per plant.

Each stem was divided longitudinally into three parts: the upper part (approximately one-fifth of the total length), the middle part (approximately three-fifths), and the lower part (approximately one-fifth, adjacent to the host root). Each part was subsequently divided transversely into the outer fraction (outer layer peeled to a depth of 0.5–2 mm, corresponding to the epidermis and the bases of the fleshy scale leaves while minimizing contamination with internal tissue) and internal tissues (Figure 7). The outer fraction ratio was calculated on a dry mass basis: outer-fraction dry mass/segment dry mass (Table A1). The dissection yielded six tissue samples per plant. Samples were sliced, flash-frozen at −80 °C, dried at 50 °C for 24 h, and stored in a glass vacuum desiccator.

### 4.2. Untargeted Metabolomics

One gram of dried tissue cut into pieces measuring approximately 1 mm was extracted in 10 mL of 70% (*v*/*v*) methanol at 4 °C for 16 h. The extract was then sonicated for 40 min and centrifuged at 12,000× *g* for 10 min at 4 °C. The resulting supernatant was filtered through a 0.22 μm PTFE membrane. The extracts were analyzed by LC-MS/MS using an Orbitrap Exploris 120 mass spectrometer (Thermo Scientific, Waltham, MA, USA) equipped with a heated electrospray ionization (H-ESI) source and CORTECS UPLC C_18_ column (1.6 μm, 2.1 × 100 mm; Waters, Milford, MA, USA). The ionization source operated in positive (spray voltage 3500 V) and negative (2500 V) modes with sheath and auxiliary gas flows of 40 and 10 Arb (sweep gas 0 Arb), an ion-transfer-tube temperature of 320 °C and a vaporizer temperature of 350 °C. The mobile phase consisted of 0.1% formic acid in water (A) and 0.1% formic acid in acetonitrile (B). The gradient was increased from 2% B to 90% B over 35 min at a flow rate of 0.3 mL/min, with a column temperature of 40 °C and an injection volume of 1 μL. Full-scan MS data were acquired over an *m/z* range of 114–1700 at a resolution of 60,000 FWHM in polarity-switching mode (AGC target 300%). Data-dependent MS/MS (Top4) used higher-energy collisional dissociation at a resolution of 15,000 FWHM with stepped normalized collision energies of 22, 24 and 28%, an isolation window of 1 *m/z*, an AGC target of 100% and a maximum injection time of 100 ms; precursors were selected using a 10 ppm mass tolerance and a minimum intensity threshold of 2000; only singly charged ions were fragmented; and dynamic exclusion was set to 30 s with isotope exclusion enabled. The 132 samples were injected in a randomized order throughout the analytical sequence. A solvent blank and a pooled QC sample were injected at the start of the sequence and after every 12 samples; ions present in the solvent blank were classified as background and excluded from subsequent analyses.

Reference standards for echinacoside, acteoside, cistanoside A, 8-epiloganic acid, geniposide, geniposidic acid, pinoresinol, pinoresinol monomethyl ether-4-O-β-D-glucoside, and syringaresinol were purchased from Shanghai Yuanye Bio-Technology Co., Ltd. (Shanghai, China). Standard solutions were analyzed using the same LC-MS/MS conditions as the tissue extracts. Features that matched the standards by retention time, accurate mass, and MS/MS fragmentation were classified as standard-confirmed; other named compounds were classified as putatively annotated. All detected features were retained for statistical analysis.

### 4.3. Data Preprocessing

Raw data were processed using Compound Discoverer software (version 3.3) with a peak-detection mass tolerance of 5 ppm, a signal-to-noise threshold of 3, and a retention-time window of 0.1 min, and processing included peak detection, retention-time alignment, adduct grouping ([M + H]^+^, [M + Na]^+^, [M + NH_4_]^+^, [M − H]^−^), compound grouping, and gap filling. Annotations were based on searches against the mzCloud, mzVault, and ChemSpider libraries together with local databases and published reports; only features for which at least one MS/MS fragment matched were retained for putative annotation; and all detected features were retained for statistical analysis.

### 4.4. Statistical Analysis

All features were normalized using probabilistic quotient normalization and log2-transformed. For multivariate analyses (PCA and OPLS-DA), the data were additionally Z-score-scaled. Features with RSD > 30% in pooled QC samples were excluded before imputation. Missing values were subsequently imputed with the Fill Gaps node in Compound Discoverer (version 3.3), which revisits the raw chromatographic data to re-search (or simulate) missing peaks by *m*/*z* and retention time; where a peak was recovered, the recovered peak area was used, and where no peak was found, the gap was filled with the detection-limit value; PCA verified QC clustering. OPLS-DA models were constructed separately for tissue type and longitudinal position and validated using 200-permutation tests. In addition to stratified seven-fold cross-validation, both models were re-validated by GroupKFold using plant identity to define the groups (all samples from each plant kept within one fold) to exclude within-plant information leakage; the grouped Q^2^ values are reported as the primary internal estimates. The contribution of each design factor was quantified using additive variance partitioning (varpart, 999 permutations), by an REML random-intercept mixed model fitted to each feature, and by Euclidean PERMANOVA (9999 permutations, within-plant permutation strata = plant). Permutation-derived R^2^ and Q^2^ intercepts were assessed to rule out overfitting, and model performance was evaluated using R^2^X, R^2^Y, and Q^2^Y; differential features were defined by OPLS-DA VIP > 1.0, |*p*(corr)| > 0.9, together with FDR < 0.05. Analysis-specific criteria are stated with the corresponding figure: the volcano filter in Figure 2D used |log2FC| ≥ 0.5 and a nominal *p* < 0.05, and the heat-map in Figure 2F used VIP > 1.9 and a nominal *p* < 1 × 10^−20^; Benjamini–Hochberg correction was applied to the model-based differential-feature set (FDR < 0.05) and to the KEGG enrichment tests (FDR < 0.05). Stratified ANOVA within each tissue–position subgroup assessed the effects of size, using summed F statistics as a composite index, restricted to confidently identified features (identification Levels 1 and 2). For Figure 1E, the 15 metabolites with the largest F-sum were selected from Level 2 identifications, and echinacoside and cistanoside A (Level 1, authentic standards) were appended as the signature phenylethanoid glycosides. Confirmatory analyses treated plant identity as a random effect: responses of key features were reassessed using linear mixed-effects models with a random intercept for plant and a Tissue × Position fixed-effect structure (Python statsmodels 0.14, MixedLM; L-BFGS with a BFGS fallback when L-BFGS did not converge), and spatial contrasts were examined using paired within-plant tests (*n* = 22). Plant-level correlations between feature abundance and continuous morphological gradients (fresh weight, stem diameter, and outer-fraction ratio) were assessed, and bootstrap resampling (1000 resamples) was used to calculate 95% confidence intervals, which are reported as the primary measure of uncertainty because they are non-parametric and robust to the modest sample size (*n* = 22); parametric Pearson *p*-values are also reported for completeness. Longitudinal position was treated as a categorical variable and was not included in these correlations. Hierarchical clustering separated samples by tissue type followed by longitudinal position. WGCNA using β = 15 (scale-free R^2^ = 0.8004, mean connectivity 15.87) resolved 10 modules (9 non-grey plus grey) among the 4309 variance-filtered metabolites, using dynamic tree cut (height 0.98), a minimum module size of 50, and eigengene merging (threshold 0.75); the unassigned grey module was omitted from subsequent enrichment analyses. Because the 132 samples were nested within 22 plants, all module–trait associations reported here were also validated using linear mixed-effects models (Section 2.6).

Module eigengenes were analyzed using linear mixed models ME ~ tissue + position + (1|plant) (lme4 1.1, lmerTest 3.2, ML estimation), reporting marginal and conditional R^2^ (MuMIn/Nakagawa convention), term-level R^2^, and a four-way variance partition (tissue, position, plant, residual; the plant share equals the ICC). Module–trait Pearson correlations are presented only as descriptive summaries. All feature-level contrasts supporting metabolite selection were therefore either (i) derived from models that included a random intercept for plant, or (ii) re-tested with such models (Section 2.5 and Section 2.6).

Counterfactual decomposition. Whole-stem relative abundances were reconstructed in linear space: log2-PQN values were back-transformed (2^x^) and averaged across the six tissue samples from each plant using their dry masses as weights (Table A1). The size-associated change was separated into a tissue-composition term (substituting the large-plant outer-fraction dry-mass share while keeping within-tissue abundances at the values for small-plant material) and an intra-tissue term (the converse); both orderings were calculated and combined using their geometric mean, which is ordering-independent and exactly satisfies the multiplicative identity for the total change. Contributions are reported as log-scale shares with plant-level bootstrap 95% CIs (1000 resamples; medians and interquartile ranges where opposing-direction components inflate the percentile interval). The decomposition is expressed in relative-abundance units and does not constitute absolute quantification (Table A2).

## 5. Conclusions

*C. deserticola* retains a clear two-axis metabolic organization despite its reduced genome and entirely subterranean growth. A radial programme (outer-fraction-dominant modules) concentrates phenylethanoid glycosides and flavonoids in the outer fraction, while internal tissues contain iridoids and primary metabolites; a longitudinal programme separates an active apical zone from a basal sugar sink. Pathway-resolved mapping showed spatial separation among the steps of this assembly route: early PhG biosynthesis proceeds throughout the stem, whereas the downstream acylation, rhamnosylation, hydroxylation and final glucosylation steps are progressively enriched in the outer fraction. The discarded outer fraction is thus consistent with a possible site of biosynthesis, rather than merely a storage site, of acteoside and echinacoside.

These findings have two implications. For parasitic-plant evolution, they caution against inferring metabolic decay from genomic streamlining [24]: spatial metabolic differentiation can persist as a developmental default even in the presence of a streamlined genome. Beyond identifying what *C. deserticola* has lost, the results indicate which organizational principles are preserved. For medicinal use, the PhG decline in larger plants occurs predominantly within tissues rather than from anatomical dilution, challenging size-based grading; retaining the outer fraction during processing and adopting tissue-aware quality standards would preserve the highest-value biosynthetic compartment.

## Figures and Tables

**Table 1:** Relationship between morphological indicators and size grades.

Size Grade	Number of Plants (*n*)	Average Weight (g)	Average Length (mm)	Average Diameter (mm)	Outer Fraction Ratio (%)	Dry Matter Ratio (%)
Small	7	151	407	23.0	29.2	29.8
Middle	9	349	446	33.2	25.7	30.5
Large	6	650	366	50.5	16.2	29.7

## Data Availability

The raw LC–MS/MS data and the processed feature table have been deposited in the China National Center for Bioinformation (CNSA) under accession number CNP0010372; the analysis code is available from the corresponding author upon request. All supplementary tables are provided with this manuscript.

## Supplementary Materials

The following supporting information can be downloaded at https://www.mdpi.com/article/10.3390/plants15213465/s1: Table S1: Metabolite annotation analysis; Table S2: Linear mixed-effects model results for the 101 hub metabolites.

## Abbreviations

The following abbreviations are used in this manuscript:

PhG(s)	Phenylethanoid Glycoside(s)
WGCNA	Weighted Gene Coexpression Network Analysis
PCA	Principal Component Analysis
OPLS-DA	Orthogonal Partial Least Squares Discriminant Analysis
KEGG	Kyoto Encyclopedia of Genes and Genomes
LC-MS/MS	Liquid Chromatography–Tandem Mass Spectrometry
QC	Quality Control
FDR	False Discovery Rate
VIP	Variable Importance in Projection
DW	Dry Weight
OF	Outer Fraction
IT	Internal Tissue
PQN	Probabilistic Quotient Normalization
REML	Restricted Maximum Likelihood
PERMANOVA	Permutational Multivariate Analysis of Variance
ICC	Intraclass Correlation Coefficient
FE	Fold Enrichment
RSD	Relative Standard Deviation

## Appendix A

**Table A1:** Morphological measurements from 22 *C. deserticola* plants.

Size Grade	Upper Diameter	Middle Diameter	Lower Diameter	Length	Mean Diameter	Fresh Weight	Upper Outer Fraction Dry Mass	Upper Internal-Tissue Dry Mass	Middle Outer Fraction Dry Mass	Middle Internal-Tissue Dry Mass	Lower Outer Fraction Dry Mass	Lower Internal-Tissue Dry Mass
	mm	mm	mm	mm	mm	g	g	g	g	g	g	g
small	24.8	18.1	19.1	350	20.67	110	4.13	5.68	5.40	12.09	2.03	5.21
	28.0	21.6	30.8	375	26.80	180	3.73	7.55	6.69	17.15	4.22	14.15
	24.4	23.4	32.7	403	26.83	170	2.17	4.73	8.68	22.94	3.98	11.50
	28.6	18.7	24.0	444	23.77	160	2.26	5.11	6.85	19.28	2.02	6.22
	21.7	21.9	25.4	361	23.00	150	2.95	5.15	6.40	16.44	3.79	7.49
	21.6	17.4	23.8	482	20.93	160	3.21	6.68	7.87	20.78	3.82	9.51
	17.9	15.6	23.6	434	19.03	130	1.69	3.19	6.78	15.78	2.85	7.72
middle	31.0	22.4	30.3	500	27.90	230	4.82	6.74	7.57	31.77	4.11	9.59
	27.5	29.8	34.9	455	30.73	260	3.86	6.76	12.22	28.21	3.88	13.19
	26.0	33.0	32.8	457	30.60	340	5.01	9.69	14.97	51.70	9.01	13.16
	29.2	29.3	44.6	488	34.37	330	5.87	8.89	12.20	43.45	3.81	12.54
	31.0	33.9	39.0	470	34.63	420	5.78	8.65	16.29	47.32	11.13	37.25
	31.9	37.0	42.8	470	37.23	500	6.99	12.70	21.98	80.73	7.05	27.34
	39.8	37.3	38.1	358	38.40	390	6.87	9.28	16.05	65.79	9.28	4.07
	28.3	38.2	42.0	345	36.17	330	7.00	16.96	16.51	61.09	4.83	12.76
	31.4	21.5	34.2	475	29.03	340	4.67	6.80	16.33	73.13	6.19	21.24
large	32.3	40.3	50.6	293	41.07	410	2.61	7.82	6.41	55.67	6.06	29.15
	44.7	49.9	66.0	440	53.53	650	5.27	19.91	16.13	134.26	5.67	37.04
	44.5	46.3	73.3	431	54.70	680	8.07	17.28	18.64	99.46	4.20	20.98
	34.9	34.9	48.9	359	39.57	970	9.80	13.78	29.90	159.53	19.54	53.19
	47.6	50.6	58.0	392	52.07	430	6.80	14.06	12.72	88.24	4.98	28.22
	48.5	63.0	75.0	279	62.17	760	7.89	16.95	16.97	115.69	9.07	48.21

**Table A2:** Counterfactual decomposition of the size-associated decline in whole-stem phenylethanoid glycoside (PhG) relative abundance. Whole-stem relative abundance was reconstructed in linear space as a dry-mass-weighted average of the six tissue samples per plant (Table A1). The large-to-small decline (total) was decomposed into a composition term (outer-fraction dry-mass share) and a within-tissue term; the composition contribution is the order-independent Shapley share (log C/log total; within-tissue share = 1 − composition share). Bootstrap 95% percentile intervals (1000 plant-level resamples). *n* denotes the number of analytical features (*m*/*z*–retention-time signals) assigned to each metabolite set; adducts were grouped during data processing (Section 4.3), so the multiple features retained per compound reflect distinct signals rather than the same ion counted twice.

Metabolite Set	*n*	Large/Small Ratio	Composition Share	95% CI	Within-Tissue Share
Echinacoside	7	0.319	38.5% †	20.4–121.2	61.5%
Acteoside	12	0.158	11.9%	8.2–17.5	88.1%
CORE (echinacoside + acteoside)	17	0.202	18.6%	12.8–27.2	81.4%
CORE + secondary PhGs	50	0.253	16.1%	12.0–21.1	83.9%
All curated PhGs	46	0.254	14.4%	10.9–19.2	85.6%

† The echinacoside 95% CI exceeds 100% because of opposing components (within-tissue term > 1 in 40/995 bootstrap draws); the bootstrap median is 38.3% (IQR 29–53%). The CORE set is the primary estimate used in the main text.
